# Utility of surveillance blood cultures in patients undergoing hematopoietic stem cell transplantation

**DOI:** 10.1186/2047-2994-3-20

**Published:** 2014-06-04

**Authors:** Sameeh S Ghazal, Michael P Stevens, Gonzalo M Bearman, Michael B Edmond

**Affiliations:** 1 Division of Infectious Diseases, Department of Internal Medicine, Virginia Commonwealth University School of Medicine, Richmond, VA, USA; 2 Research and Scientific Publication Center, King Fahad Medical City, Children’s Specialized Hospital, P O box: 59046, Riyadh 11525, Saudi Arabia

## Abstract

**Background:**

Surveillance blood cultures are often obtained in hematopoietic stem cell transplant (HSCT) patients for detection of bloodstream infection. The major aims of this retrospective cohort study were to determine the utility of the practice of obtaining surveillance blood cultures from asymptomatic patients during the first 100 post-transplant days and to determine if obtaining more than one positive blood culture helps in the diagnosis of bloodstream infection.

**Methods:**

We conducted a 17-month retrospective analysis of all blood cultures obtained for patients admitted to the hospital for HSCT from January 2010 to June 2011. Each patient’s clinical course, vital signs, diagnostic testing, treatment, and response to treatment were reviewed. The association between number of positive blood cultures and the final diagnosis was analyzed.

**Results:**

Blood culture results for 205 patients were reviewed. Cultures obtained when symptoms of infection were present (clinical cultures) accounted for 1,033 culture sets, whereas 2,474 culture sets were classified as surveillance cultures (no symptoms of infection were present). The total number of positive blood cultures was 185 sets (5.3% of cultures obtained) and accounted for 84 positive culture episodes. Incidence of infection in autologous, related allogeneic and unrelated allogeneic transplants was 8.3%, 20.0%, and 28.6% respectively. Coagulase-negative staphylococci were the most common organisms isolated. Based on our application of predefined criteria there were 29 infections and 55 episodes of positive blood cultures that were not infections. None of the patients who developed infection were diagnosed by surveillance blood cultures. None of the uninfected patients with positive blood cultures showed any clinical changes after receiving antibiotics. There was a significant difference between the incidence of BSI in the first and second 50-day periods post-HSCT. There was no association between the number of positive blood cultures and the final diagnosis.

**Conclusion:**

Surveillance blood cultures in patients who have undergone HSCT do not identify bloodstream infections. The number of positive blood cultures was not helpful in determining which patients had infection. Patients are at higher risk of infection in the first 50 days post-transplant period.

## Introduction

Bloodstream infection (BSI) remains a significant complication following hematopoietic stem cell transplantation (HSCT), with post-transplant BSI occurring in 12.5 to 41% of patients [1-8]. Antimicrobial prophylaxis and corticosteroid therapy may make it more difficult to diagnose BSI in patients with HSCT as the yield of blood cultures in patients who are already on antibiotics is limited [9-11] and corticosteroids may mask the signs of inflammation [12]. For these reasons, some experts have recommended surveillance blood cultures to detect occult bacteremia [12]. Previous studies regarding the utility of surveillance blood cultures in patients undergoing hematopoietic stem cell transplantation (HSCT) are scarce with small numbers of patients, and describe specific subgroups of patients in relation to the type of transplant or the patients’ age [13-17]. Moreover, these studies have come to differing conclusions [10-13].

The primary objective of this study was to investigate whether surveillance blood cultures (performed weekly per our hospital protocol) are valuable in the early diagnosis of BSI during the first 100 days post-transplant. Secondary objectives were: to determine whether the frequency of infection differs between the first 50 days post-transplant and the second 50 days and to identify the relevance of the number of positive cultures sets in the diagnosis of bloodstream infection.

## Methods

A retrospective cohort study was performed at an 820-bed academic medical center in an attempt to understand why high rates of contaminated blood cultures were observed in the HSCT population. Since this project was considered to be a quality improvement study, institutional review board approval was not obtained. Permission was received from the hospital to use the patient data for this report.

Using the BMT unit log book all patients who underwent HSCT in the 17-month period between January 1, 2010 and May 31, 2011 were identified. Electronic medical records of these patients were reviewed and all blood culture results identified. Standard practice was to obtain all blood samples for blood cultures from central lines; obtaining concomitant blood samples from a peripheral vein was not routinely performed.

Each patient’s clinical course, vital signs, diagnostic testing, treatment, and response to treatment were reviewed for one week before the date of each positive blood culture until discontinuation of antibiotics (if given), hospital discharge or death. Findings were tabulated and evaluated against clinical diagnostic criteria established a priori.

### Definitions

#### Blood cultures

A surveillance blood culture was defined as a blood culture done at a regular interval of at least 5 days (weekly as per our hospital protocol or 5 days if the screening culture due time is on the weekend) in the absence of clinical features of infection (fever, hypotension, or malaise). A clinical blood culture was defined as a blood culture done in the presence of systemic symptoms of infection (fever, hypotension, and/or malaise) and/or a blood culture obtained <5 days after a previous blood culture as the surveillance blood culture definition required a 7-day interval between cultures (5 days if due on the weekend).

### Outcomes

Each positive blood culture was evaluated and a determination made as to whether it represented infection or not. With absence of a gold standard for the clinical diagnosis of catheter-related bloodstream infection, we utilized the CDC criterion: the patient has at least one of the following signs or symptoms: fever (>38°C), chills, or hypotension, and the signs and symptoms and positive laboratory results are not related to an infection at another site. Bloodstream infection was defined as one or more positive blood cultures obtained in the presence of clinical features with systemic antibiotics administered for more than 5 days [4,6,9,10]. Uninfected patients were defined as those who had one or more positive blood cultures but no clinical features of infection. A positive culture episode was defined as one positive blood culture set or group of successive positive blood cultures sets all done within one week, irrespective of their number or whether the patient was classified as infected or not. Clinical features were defined as fever (temperature >38°C), and/or hypotension (systolic blood pressure less than 90 mm Hg or diastolic less than 60 mm Hg), and/or malaise.

### Statistical methodology

Proportion of positive cultures was compared using the Chi square test or Fisher’s exact test when appropriate. The one-sample proportion t-test was used to compare proportions of infections in the first and last 50 days follow up period. The level of significance (α) was set at 0.05. SAS software was used for all statistical analyses.

## Results

**Figure 1:**
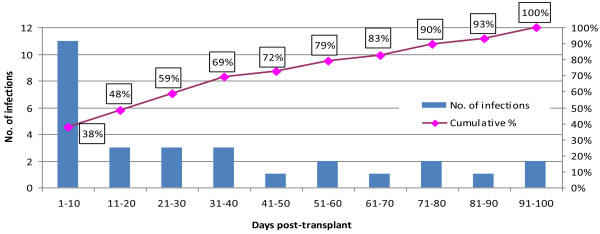
Timeline of infections.

The number of patients who underwent HSCT during the study period was 213. Data could not be retrieved for 8 patients; therefore the medical records of 205 patients were reviewed. For these patients, the total number of blood cultures obtained was 3,507 culture sets (mean 17 cultures per patient; range 1–60). Clinical cultures accounted for 1,033 cultures and 2,474 were classified as surveillance cultures. The total number of positive blood cultures was 158 (4.5% of cultures obtained) and accounted for 84 positive culture episodes. The incidence of bloodstream infection in autologous, related allogeneic and unrelated allogeneic transplants was 8.3%, 20.0%, and 28.6% respectively (Table 1).None of the patients who developed bloodstream infection were diagnosed by surveillance blood cultures. Based on our predefined criteria there were 29 infections and 55 episodes of positive blood cultures that were not infections. All patients in the infection group and 21 patients in the non-infection group received systemic antibiotics for at least 10 days per episode. None of the 21 uninfected patients had symptoms before the surveillance culture or showed any clinical changes after receiving antibiotics. The majority of infections [21/29 (72%)] occurred in the first 50 days post-transplant (p = 0.008), and nearly half occurred in the first 20 days (Figure 1).

**Table 1:** Incidence of bloodstream infection in relation to type of HSCT

**Type of HSCT**	**Infection**	**No infection**	**Patients**	**Incidence of infection**
Autologous	11	9	133	8.3%
Allogeneic (related)	6	21	30	20.0%
Allogeneic (unrelated)	12	25	42	28.6%
Total	29	55	205	14.1%

The most common organisms isolated were coagulase-negative staphylococci, 41/84 (49%) in pure culture and an additional 10 (12%) in mixed cultures. *Klebsiella spp.* and enterococci each accounted for 5 episodes (6%) of positive cultures; viridans group streptococci accounted for 2 episodes (2.4%); and the remaining 25% were caused by different organisms each occurring only once (Table 2).

**Table 2:** Organisms detected in 84 positive blood culture episodes

**Organism**	**Diagnosis**		**Total**
	**Infection**	**Non-infection**	
Coagulase-negative staphylococci	12 (41%)	29 (52.7%)	41 (48.8%)
Mixed (any two organisms)	03 (10%)	07 (12.7%)	10 (11.9%)
*Klebsiella* spp	03 (10%)	02 (03.6%)	5 (6.0%)
Enterococci	03 (10%)	02 (03.6%)	5 (6.0%)
Viridans group streptococci	00	02 (03.6%)	2 (2.4%)
Other	08* (28%)	13+ (23.6%)	21 (25.0%)
Total	29 (100%)	55 (100%)	84 (100.0%)

*Include one Yeast.

+Include two Candida species.

The clinical feature most common in infected patients was fever (22/29 [41%]); malaise occurred in 12/29, and hypotension in 3/29 infections. Some patients had combinations of the above three signs and symptoms. Importantly, none of the 29 patients with the final diagnosis of infection were diagnosed by surveillance cultures.

BSIs were more frequent in the first 50 days following transplant (p = 0.008). There was no statistically significant difference between the proportion of BSIs diagnosed by one positive blood culture or more than one positive blood culture (p = 0.40). This was also true for the subset of BSIs due to coagulase-negative staphylococci (Tables 3 and 4).

**Table 3:** Number of positive blood culture sets in infection and non-infection episodes (all organisms)

	**Number of positive blood cultures**		
**Diagnosis**	**1**	**>1**	**Total**
Infection	23 (79.3%)	6 (20.7%)	29
No Infection (contamination/colonization)	39 (70.9%)	16 (29.1%)	55
Total	62 (83.8%)	22 (16.2%)	84

P = 0.40.

**Table 4:** Number of positive blood culture sets in infection and non-infection episodes due to coagulase-negative staphylococci

	**Number of positive blood cultures**		
**Diagnosis**	**1**	**>1**	**Total**
Infection	12 (100.0%)	0 (0.0%)	12 (100%)
No infection (contamination/colonization)	26 (89.7%)	3 (10.3%)	29 (100%)
Total	38	3	41

## Discussion

BSI occurred in 14.1% of our patients, which is near the lower limits of the previously published rates (12.5-40%) [1-8]. Patients undergoing unrelated allogeneic HSCT had the highest incidence of infection (29%), followed by related allogeneic HSCT (20%), and lowest in autologous HSCT (8%).

Studies addressing the utility of surveillance blood cultures in HSCT patients are few, even though performance of surveillance blood cultures is standard practice at many centers. In non-immunosuppressed patients, surveillance cultures are not performed since nearly all positive cultures will represent either contamination, or in the case of cultures drawn through indwelling central venous lines, colonization of the catheter lumen. On reviewing the medical records of our 205 HSCT patients with predefined diagnostic criteria, we found that surveillance blood cultures did not detect a single case of BSI. Thus, the performance of surveillance blood cultures resulted in over diagnosis and subsequent treatment for 21 patients in our cohort. This drives the overuse of antibiotics, which may result in increasing rates of antibiotic resistance, increased cost, and potential complications such as *Clostridium difficile* infection.

Chizuka et al. [13] described 15 of 25 (60%) patients on corticosteroid therapy following allogenic HSCT that had definitive bloodstream infection without fever at the time of diagnosis; moreover, 4 of them remained afebrile during the course of infection. Diagnostic criteria for bloodstream infection in this paper were the presence of 2 positive blood cultures for common skin contaminants or one positive culture for other pathogens, but clinical findings were not included as part of the diagnostic criteria. This might lead to misclassifying colonized central lines as BSI (i.e., over diagnosis). Frere et al. [14] concluded that routine surveillance cultures predicted bacteremia in their cohort of 505 HSCT patients; however, there was no clear case definition for BSI. Moreover, they refer as well to their finding that for coagulase-negative staphylococci, the predictive value of surveillance cultures is quite low because of the very high rate of central venous line colonization observed in their patients (77%). They also found that routine surveillance blood cultures were unable to detect bacteremia with streptococci and anaerobes.

Penack et al. [15] detected microbial growth prior to the onset of infection symptoms in 3 of 45 neutropenic episodes in 39 patients that led to modifications in patient management, but they did not describe their case definition. They noted that shortly after starting antibiotics, the three patients showed signs of infection; thus, it is possible that the blood cultures detected line colonization that preceded infection. Rigby et al. [16] reported that 3 of 43 positive blood cultures in neutropenic children which yielded organisms, when repeated prior to starting antibiotics were sterile, suggesting that these positive cultures represented colonization rather than infection. Czirok et al. [17] found that in 14 episodes of fever in 23 HSCT patients*,* surveillance cultures yielded bacteria identical with those in clinical blood cultures in only one episode and in the remaining three documented BSIs the identified bacteria isolated by surveillance cultures was not the same as the bacteria identified by clinical blood cultures. They concluded that surveillance blood cultures were of limited value in predicting infection. Neither this study nor any one of the studies mentioned above utilized differential time to positivity as did Abdelkafi et al. [18], who found that marker was associated with 86% sensitivity and 87% specificity. Thus it seems that surveillance blood cultures result in identification of central line colonization status, which does not require treatment.

We found that bloodstream infections were more frequent in the early post-transplant period. We also found that there was no statistically significant difference between BSI diagnosed by one positive blood culture or more than one positive blood culture, and this implies that the current CDC surveillance definition for central line associated bloodstream infection that requires at least two positive blood cultures for common skin contaminants such as coagulase-negative staphylococci may not be useful in this patient population and should be further evaluated in other studies.

The strengths of our study are the relatively large number of patients evaluated (205), and the inclusion of patients of all age groups and all types of HSCT. A weakness of the study is that it was performed at a single center and there may be local institutional practices that could limit the generalizability of our results. In addition, retrospective review of clinical records for symptoms of infection could have resulted in misclassification of infected patients as uninfected, though including vital signs in the definition, which were consistently recorded for these patients, should limit the degree of misclassification.

## Conclusions

Patients undergoing HSCT are at a high risk of bloodstream infection, especially those patients undergoing unrelated allogeneic transplantation, and coagulase-negative staphylococci were the most common cause. We demonstrate that post-HSCT surveillance blood cultures are not useful for the early detection of bacteremia and likely result in increased cost and unnecessary use of antibiotics. Post-HSCT patients have higher risk of infection during the first 50 days post-transplant. Lastly, the number of positive blood cultures was not useful in the determination of infection in these patients.

## Competing interests

The authors declare that they have no competing interests.

## Authors’ contributions

SSG: Collecting data, Data analysis, writing the draft. MPS: Search internet, summarizing evidence, revising the MS. GMB: Search internet, summarizing evidence, revising the MS. MBE: Leading the team, supervising the work, directing and giving advice, revising the manuscript and approval person. All authors read and approved the final manuscript.
